# Supplementary material for: WordCommentsAnalyzer: A windows software tool for qualitative research
Source: F1000Res. 2018 Sep 4;7:536. Originally published 2018 May 3. [Version 2] doi: 10.12688/f1000research.14819.2 (PMC6137411; doi:10.12688/f1000research.14819.2)
Supplement: Supplementary file 3 [file f1000research-7-17614-s0002.tgz › b2a79496-4ada-47c4-9682-a99ce867836d.zip!/Eight Iranian Health Journals/Yafteh _1.docx]

© Yafteh  . This work is licensed under CC BY 4.0 (https://creativecommons.org/licenses/by/4.0/).

Volume & Issue: دوره 19 - شماره 5

Title: بررسی عملکرد ماده خشک و بازده اسانس در سه گونه مرزهSatureja mutica ,Satureja rechingeri و Satureja khuzestanicaدر شرایط اقلیمی شهر خرم آباد

http://yafte.lums.ac.ir/article-1-688-fa.html

مقدمه: این مطالعه به منظور بررسی عملکرد ماده خشک و بازده اسانس بر روی 8 اکسشن از سه گونه مرزه آزمایشی در ایستگاه تحقیقات کشاورزی شهر خرم آباد انجام شد. مواد و روش‌ها: بذور هر یک از اکسشن ­ها در سال 1389 داخل گلدان کشت شدند و پس از 16-14 برگه شدن به داخل زمین انتقال یافتند و در قالب طرح بلوک های کامل تصادفی در 3 تکرار کشت شدند. سپس عملکرد خشک، درصد اسانس و عملکرد اسانس در هکتار اندازه‌ گیری شد. پس از جمع آوری داده­ ها تحلیل واریانس یک طرفه و مقایسه میانگین ­ها به روش دانکن انجام شد. یافته ­ها: نتایج تحلیل واریانس نشان داد که بین گونه­ ها و بین اکسشن­ های داخل گونه­ ها از نظر کلیه صفات ارزیابی شده در سطوح احتمال 1 درصد اختلاف معنی دار بود. در بین گونه­ ها، گونه  S. mutica و در بین اکسشن­ ها، اکسشن شماره 2 از گونه  S. muticaاز نظر صفات مورد مطالعه برتری بهتری نشان دادند. بحث و نتیجه ­گیری: نتایج بدست آمده در این تحقیق نشان داد که عملکرد ماده خشک و  بازده اسانس گونه S. mutica  از دو گونه   S.rechingerو S. Khuzestanica بیشتر بوده و به عنوان یکی از گونه­ های مناسب برای اهلی کردن کشت و کار در منطقه خرم آباد پیشنهاد می ­شود.

Title: رابطه راهبردهای یادگیری و خود کارآمدی تحصیلی با پیشرفت تحصیلی دانشجویان دانشگاه علوم پزشکی لرستان در سال تحصیلی 1395

http://yafte.lums.ac.ir/article-1-2558-fa.html

مقدمه: این پژوهش با هدف بررسی رابطه راهبردهای یادگیری و خود کارآمدی تحصیلی با پیشرفت تحصیلی دانشجویان دانشگاه علوم پزشکی لرستان در سال تحصیلی 1395 انجام شد. مواد و روش‌ ها: این مطالعه توصیفی تحلیلی از نوع همبستگی بود. جامعه آماری شامل کلیه دانشجویان دانشگاه علوم پزشکی لرستان (2200 نفر) بودند. حجم نمونه با استفاده از جدول کرجسی و مورگان 330 نفر تعیین شد که به روش نمونه‌گیری تصادفی طبقه‌ای­ نسبی انتخاب شدند. ابزار گردآوری داده‌ها پرسشنامه‌های استاندارد راهبردهای یادگیری پینتریچ و دیگروت، خود کارآمدی تحصیلی جیکنز و مورگان و معدل دانشجویان بود. داده‌ها با استفاده از ضریب همبستگی پیرسون و تحلیل رگرسیون به روش گام به گام تجزیه و تحلیل شدند. یافته ­ها: نتایج نشان داد که رابطۀ مثبت و معنا‌داری بین راهبردهای یادگیری و خود کارآمدی تحصیلی با پیشرفت تحصیلی دانشجویان وجود دارد. همچنین نتایج رگرسیون نشان داد که راهبردهای یادگیری و ابعاد خود کارآمدی تحصیلی حدود 12 درصد نمرات پیشرفت تحصیلی دانشجویان را تبیین می­کنند. بحث و نتیجه ­گیری: نتایج این پژوهش بیانگر تأثیر چشمگیر راهبردهای یادگیری (شناختی و فراشناختی) و خود کارآمدی تحصیلی بر پیشرفت تحصیلی دانشجویان بود.

Title: رابطه بین مصرف دوز مزمن متامفتامین بر روی هورمون‌های تستوسترون،LH و FSH در مردان

http://yafte.lums.ac.ir/article-1-2364-fa.html

مقدمه: متامفتامین به سادگی از مواد شیمیایی در دسترس مثل پسودوافدرین ساخته می ­شود. این دارو که قبلاً در درمان ADHD به کار برده می ­شد، سهم عمده­ای از کنترل اعمال جنسی در مردان و زنان از طریق ترشح هورمون گنادوتروپین GNRH از هیپوتالاموس دارد. این هورمون از طریق عروق پورت وارد هیپوفیز قدامی شده و موجب ترشح هورمون LH و FSH  می­شود. هدف از این پژوهش بررسی رابطه بین مصرف دوز مزمن متامفتامین بر روی هورمون­های تستوسترون، LH و FSH در مردان می ­باشد. مواد و روش‌ها: در این مطالعه 30 نفر از مردان مصرف کننده دوز مزمن متامفتامین مراجعه کننده به کلینیک ­های درمانی در سطح شهر اصفهان با استفاده از روش نمونه‌ گیری تصادفی انتخاب و مورد سنجش قرار گرفتند. افراد مصرف کننده ابتدا پرسشنامه مقیاس شدت اعتیاد (ASI) را پر نموده و پس از خون­گیری، نمونه ­ها به آزمایشگاه منتقل شد. یافته­ ها: نتایج نشان داد که مدت مصرف متامفتامین بر روی میزان هورمون FSH تأثیر ندارد. تفاوت معنی­داری بین زمان­های مختلف مشاهده نگردید. ولی مدت مصرف متامفتامین بر روی میزان هورمون LH و تستوسترون تأثیر دارد. بحث و نتیجه ­گیری: مدت مصرف متامفتامین بر روی میزان هورمون LH و تستوسترون تأثیر داشته ولی بر روی هورمون FSH تأثیر ندارد.

Title: تأثیر بازی‌های پرورشی بر اختلالات رفتاری درونی و بیرونی دختران پیش‌دبستانی

http://yafte.lums.ac.ir/article-1-2316-fa.html

مقدمه: اختلالات رفتاری کودکان پیش‌دبستانی مشکلات عدیده‌ای ایجاد می‌کند. برای رفع این مشکل به‌طور عمده از درمان دارویی استفاده می‌شود، لیکن این تحقیق به دنبال جایگزین غیر دارویی از طریق بازی‌های پرورشی است. مواد و روش‌ها: از طریق روش پژوهش تصادفی کارآزمایی کنترل شده با استفاده از طرح گروه کنترل نابرابر و پیش آزمون-پس آزمون اقدام به گردآوری اطلاعات گردید. نمونه آماری شامل 46 دختر 6 ساله بود. آزمودنی‌ها از میان یک مهدکودک دخترانه شهر بروجرد انتخاب و از طریق ایجاد توالی تصادفی (24 نفر گروه تجربی و 22 نفر گروه کنترل) در دو گروه جایگزین شدند. ابزار تحقیق فرم مشاهده (PKBS) برای سنجش اختلالات رفتاری است. داده‌های حاصل از طریق آزمون تی مستقل و وابسته تحلیل شد. یافته­ ها: نتایج نشان داد بین میانگین پیش آزمون و پس آزمون مشکلات رفتاری درونی (001/0≥P) و بیرونی (001/0≥P) دختران پیش‌دبستانی گروه تجربی تفاوت معنی‌داری وجود دارد. نتایج آزمون تی مستقل نشان می‌دهد که بین پس آزمون مشکلات رفتاری درونی (05/0P<) گروه تجربی و کنترل و بیرونی آنها (01/0P<) تفاوت معنی‌داری به نفع گروه تجربی وجود دارد. بین میانگین پیش آزمون و پس آزمون مشکلات رفتاری درونی (69/0P=) و بیرونی (23/0P=) گروه کنترل تفاوت معنی‌داری مشاهده نشد. بحث و نتیجه ­گیری: بازی‌های پرورشی تأثیر معنی‌داری بر بهبود اختلالات رفتاری دختران پیش‌دبستانی دارد. لذا مناسب است در مراکز مربوط برای کودکانی که اختلالات رفتاری دارند، بازی‌های پرورشی به عنوان یک برنامه درمانی غیر دارویی اعمال گردد.

Title: مقایسه ویژگی های شخصیتی افراد مبتلا به سندروم روده تحریک‌پذیر و افراد سالم: مدل پنج عاملی

http://yafte.lums.ac.ir/article-1-2422-fa.html

مقدمه: سندروم روده تحریک‌ پذیر از بیماری­ های گوارشی شایعی است که طبق مدل زیستی-روانی-اجتماعی، ویژگی­ های شخصیتی نقش عمده­ای در شروع، عود و وخامت آن ایفا می­کند. هدف پژوهش حاضر مشخص کردن ویژگی­ های شخصیتی افراد مستعد سندروم روده تحریک‌ پذیر بود. مواد و روش‌ها: در این مطالعه ­ی مقطعی تحلیلی، ویژگی­ های شخصیتی 50 نفر از بیماران مبتلا به سندروم روده تحریک که بیماری‌شان توسط متخصص گوارش تأیید شده بود، با 50 نفر از افراد سالم مقایسه شد. داده­ های پژوهش از طریق پرسشنامه ­های شخصیت پنج عاملی نئو (NEO-FFI) گردآوری و با روش آماری تحلیل واریانس چند متغیره مورد تجزیه و تحلیل قرار گرفت. یافته ­ها: یافته ­های پژوهش نشان داد که افراد مبتلا به سندروم روده تحریک‌ پذیر از روان آزرده­خویی بیشتر و برون­گرایی کمتری نسبت به افراد سالم برخوردارند (05/0>P) و از نظر سایر ویژگی ­های شخصیتی، تفاوت معناداری بین این دو گروه مشاهده نشد. بحث و نتیجه­گیری: یافته ­های پژوهش بیانگر این بود که برخی ویژگی­های شخصیتی، افراد را مستعد آسیب‌ پذیری بیشتر نسبت به سندروم روده تحریک‌ پذیر می­کند و بر اساس ویژگی­ های شخصیتی می­توان احتمال ابتلا به سندروم روده تحریک‌ پذیر را پیش­بینی کرد.

Title: فراوانی ژن‌های بتالاکتاماز طیف گسترده در ایزوله‌های اشریشیاکلی جدا شده از بیماران سرپایی مبتلا به عفونت ادراری در استان گیلان

http://yafte.lums.ac.ir/article-1-2402-fa.html

مقدمه: اشریشیاکلی رایج ‌ترین عامل عفونت ادراری می‌باشد. مقاومت به سفالوسپورین‌ها به دلیل تولید بتالاکتامازهای طیف گسترده (ESBLs) اخیراً افزایش یافته است. هدف از انجام این مطالعه بررسی فراوانی ژن‌های CTX-M، SHV، TEM، OXA-1، PER-2 و VEB-1 در اشریشیاکلی جدا شده از نمونه‌های ادراری بیماران سرپایی مبتلا به عفونت ادراری در گیلان بود. مواد و روش‌ها: تعداد 2267 نمونه ادرار در طول شش ماه از آزمایشگاه‌های منتخب گیلان از بیماران سرپایی جمع‌آوری شد. ابتدا با تست‌های بیوشیمیایی و افتراقی مثل MRVP و دکربوکسیلاسیون لیزین در محیط LIA، هیدرولیز اوره و TSI، تولید اوره و سیمون‌سیترات، ایزوله‌های اشریشیاکلی شناسایی شدند. جهت تعیین مقاومت‌دارویی از انتشار دیسک و برای تولید ESBL، از دیسک دوتایی با استفاده از دیسک سفتریاکسون، آموکسی‌کلاو و سفتازیدیم-کلاولونات استفاده شد. بررسی ژن‌های مورد مطالعه در ایزوله‌ها با PCR و پرایمرهای اختصاصی هر ژن انجام گرفت. یافته ­ها: از 2267 نمونه، تعداد 167 ایزوله اشریشیاکلی شناسایی گردیدند. بر اساس روش دیسک دوتایی، 9/38% ESBL مثبت بودند. بررسی مولکولی نمونه‌ها نشان داد که میزان 32/70% ژن CTX-M، 64/9% ژن TEM، 88/4% ژن SHV، 02/57% ژن OXA-1 و 01/12% ژن PER-2 را دارا هستند، اما VEB-1 یافت نشد. تعدادی از ایزوله‌ها نیز دارای چندین ژن مقاومت به‌ طور همزمان بودند. از نظر آماری بین جنس و سن با این بیماری و همچنین بین توانایی تولید ESBL و وجود ژن‌های مورد بررسی رابطه مستقیم وجود دارد (05/0P<). بحث و نتیجه ­گیری: در مطالعه حاضر بیش از نیمی از ایزوله‌های اشریشیاکلی مولد ESBL بودند. با توجه به اینکه عوامل مقاومت روی عناصر ژنتیکی متحرک قرار دارند، شناسایی سریع این سویه‌ها می‌تواند نقش مهمی در جلوگیری از گسترش آن‌ها داشته باشد.

Title: بررسی تأثیر مکمل زنجبیل بر بیان ژن آنزیم‌های سوپراکسید دیسموتاز، کاتالاز و گلوتاتیون پراکسیداز و سطح پراکسیداسیون لیپیدی در مردان سالم

http://yafte.lums.ac.ir/article-1-2440-fa.html

مقدمه: هدف از تحقیق حاضر بررسی تأثیر مکمل زنجبیل بر بیان ژن آنزیم‌های کاتالاز (CAT)، گلوتاتیون پراکسیداز (GPX)، سوپراکسید دیسموتاز (SOD) و سطوح مالون دی آلدهید (MDA) بود. مواد و روش‌ها: در یک کارآزمایی نیمه تجربی، از میان دانشجویان پسر 25-20 ساله دانشگاه شاهد، 20 نفر انتخاب و‌ به‌ طور تصادفی به دو گروه تجربی (10 نفر) و کنترل (10 نفر) تقسیم شدند. آزمودنی‌های گروه تجربی روزانه یک گرم کپسول زنجبیل را در دوزهای 250 میلی‌گرمی، 4 بار در روز و به مدت 8 هفته دریافت کردند. افراد گروه کنترل نیز دارونما (مالتودکسترین طعم داده شده) را به همین شکل دریافت کردند. نمونه‌های خونی 72 ساعت قبل از شروع و 72 ساعت بعد از اتمام پروتکل تحقیق، گرفته شد. از روش‌های آماری کولموگروف-اسمیرنوف، t همبسته و مستقل و همچنین نرم‌افزار SPSS (نسخه 22) و اکسل استفاده شد. همچنین سطح معنی‌داری کمتر از 05/0 در نظر گرفته شد. یافته ­ها: بین گروه‌ها تفاوتی در بیان ژن آنزیم‌های آنتی‌اکسیدانی و سطوح MDA دیده نشد (144/0P= برای SOD، 234/0P= برای CAT، 1/0P= برای GPX و 201/0P= برای MDA). در گروه تجربی، مکمل زنجبیل تنها باعث کاهش معنادار MDA شد (01/0P=). بحث و نتیجه ­گیری: با توجه به نتایج این تحقیق می‌توان گفت 8 هفته مصرف مکمل زنجبیل احتمالاً می‌تواند بدون تأثیر گذاشتن بر بیان ژن آنزیم‌های آنتی‌اکسیدانی، پراکسیداسیون لیپیدی را کاهش دهد.

Title: ردیابی فنوتیپی و مولکولی ژن‌های مولد بتالاکتاماز وسیع‌الطیف CTX-M در جدایه‌های اشریشیاکلی از طیور مبتلا به کلی‌باسیلوز در شهرستان خرم‌آباد

http://yafte.lums.ac.ir/article-1-2391-fa.html

مقدمه: اکثر سویه‌های اشریشیاکلی دارای مقاومت چندگانه دارویی بوده و این باکتری عامل اصلی بیماری کلی‌باسیلوز طیور است، صدمات اقتصادی عفونت‌های حاصل از E.coli خصوصاً در صنعت طیور بسیار بالا بوده و سالانه هزینه‌های زیادی صرف درمان بیماری‌های ناشی از آن با آنتی‌بیوتیک‌ها می‌شود، لذا هدف از این مطالعه ارزیابی حضور ژن bla-CTX-M در جدایه‌های اشریشیاکلی به دست آمده از نمونه‌های مرضی طیور می‌باشد. مواد و روش‌ها: در این مطالعه 100 جدایه باکتری اشریشیاکلی از اندام‌های مختلف طیور مبتلا به کلی‌باسیلوز جداسازی گردید و با استفاده از تست‌های بیوشیمیایی مورد تأیید قرار گرفت، جدایه‌ها از نظر حضور ژن‌های CTX-M به روش PCR بررسی شدند. یافته‌ها: از 100 جدایه مورد بررسی 4 جدایه (4%) به صورت ESBL مثبت بودند، از این تعداد نیز 25% دارای ژن CTX-M بودند. بحث و نتیجه‌ گیری: با توجه به اهمیت اشریشیاکلی در عفونت‌های طیور و همچنین شیوع بالای سویه‌های تولیدکننده بتالاکتاماز وسیع‌الطیف باید از روش‌های تشخیصی سریع در تعیین این سویه‌ها در آزمایشگاه‌ها به صورت روتین استفاده گردد. نتایج حاصل از این مطالعه نشان می‌دهند که اشریشیاکلی کومنسال دستگاه گوارش طیور به عنوان مخزنی برای بتالاکتامازهای وسیع‌الطیف بوده که این موضوع از لحاظ بهداشت عمومی و انتقال مقاومت‌های آنتی‌بیوتیکی به انسان دارای اهمیت می‌باشد.

Title: مقایسه تاب‌آوری، سبک زندگی و کیفیت زندگی در بین بیماران قلبی عروقی و افراد سالم

http://yafte.lums.ac.ir/article-1-2429-fa.html

مقدمه: امروزه بیماری‌های قلبی عروقی به عنوان مهم‌ترین عامل مرگ‌ ومیر در جهان شناخته شده است. پژوهش حاضر با مقایسه تاب‌ آوری روانی، سبک زندگی و کیفیت زندگی در بین بیماران قلبی- عروقی و افراد سالم انجام شده است. مواد و روش­ ها: پژوهش حاضر از نوع علتی- مقایسه‌ ای است. جامعه مورد مطالعه شامل کلیه افراد دارای بیماری قلبی- عروقی بیمارستان شهید مدنی خرم‌آباد بودند. 196 نفر بیماران قلبی- عروقی با استفاده از روش نمونه‌ گیری در دسترس انتخاب و با 196 نفر افراد سالم همتاسازی شده، مقایسه گردیدند. برای اندازه‌گیری متغیرهای پژوهش از آزمون‌های تاب‌آوری روانی (کونور و دیویدسون، 2003)، سبک زندگی (لعلی و همکاران، 1391) و کیفیت زندگی (سازمان بهداشت جهانی، 1996) استفاده شد. داده‌ها توسط آزمون یومان ویتنی و با سطح اطمینان 95% و با استفاده از نرم افزار SPSS نسخه 20 در دو سطح توصیفی و استنباطی تجزیه و تحلیل شدند. یافته ­ها: بین سبک زندگی و مؤلفه‌های آن، همچنین بین کیفیت زندگی و مؤلفه وضعیت کلی سلامتی، حیطه سلامت جسمانی، حیطه روانشناختی، حیطه محیط زندگی و تاب‌ آوری روانی در بیماران قلبی- عروقی و افراد سالم تفاوت معناداری وجود داشت. بحث و نتیجه­ گیری: با توجه به اینکه بیماران قلبی- عروقی نمرات پایین‌تری را نسبت به افراد سالم در شاخص‌های تاب‌آوری، سبک زندگی و کیفیت زندگی به دست آوردند، به نظر می‌رسد که احتمالاً تاب‌آوری و سبک زندگی خاص و کیفیت زندگی ویژه آنها در زمینه سازی بیماری یا حفظ علائم مؤثر می‌باشد و در زمان درمان بیماران می‌تواند مورد توجه قرار گیرد.

Title: پیش‌بینی سلامت روانی بر اساس استحکام روانی با واسطه‌گری ابعاد شخصیت

http://yafte.lums.ac.ir/article-1-2365-fa.html

مقدمه: امروزه با افزایش فشارهای زندگی و به سبب آن فشار روانی، سلامت روانی افراد به مخاطره افتاده است. در همین راستا، می‌توان به سبک شخصیتی و میزان استحکام روانی افراد اشاره کرد که بر سلامت روان تأثیرگذار است. هدف این پژوهش بررسی نقش پیش‌بینی کننده استحکام روانی در سلامت روان به دو شکل مستقیم و غیرمستقیم با میانجی‌گری ابعاد شخصیت خواهد بود. مواد و روش‌ها: در این مطالعه‌ای مقطعی 273 دانشجوی پردیس مرکزی دانشگاه علامه طباطبایی در سال تحصیلی 93-92 به روش نمونه‌گیری خوشه‌ای انتخاب شدند. ارزیابی متغیرها با استفاده از پرسشنامه‌های سلامت عمومی، شخصیت پنج عاملی نئو و استحکام روانی کلاف انجام شد. داده‌ها با روش مدل یابی تحلیل مسیر و با استفاده از نرم‌افزارهای SPSS 21 و LISREL تحلیل شد. یافته‌ ها: مجذور خی مدل فرضی برابر با 47/2 به دست آمد، این شاخص با سطح معناداری 116/0 غیر معنادار بوده که بیانگر متناسب و برازنده بودن مدل هست. شاخص‌های برازندگی دیگر 98/0=GFI، 97/0=NFI، 97/0=CFI، 93/0=AGFI و 06/0=RMSEA  است که میزان قابل‌قبول مدل را داراست. متغیر تعهد، کنترل زندگی و اعتماد به نفس در روابط بین فردی به‌صورت غیرمستقیم توانستند سلامت روان را پیش‌بینی نمایند (05/0P<) و کنترل هیجان و اعتماد به نفس در روابط بین فردی به‌صورت مستقیم توانستند سلامت روان را پیش‌بینی نمایند (05/0P<). بحث و نتیجه‌ گیری: برخی ویژگی‌های استحکام روانی فارغ از اثر شخصیت می‌توانند موجب سلامت روانی شوند که طبق یافته‌ها کنترل هیجان و اعتماد به نفس از اهمیت بیشتری برخوردارند. پیشنهاد می‌شود برای بهبود سلامت روانی این عوامل مورد آموزش قرار گیرند.

Title: مطالعه توزیع پلی‌مورفیسم‌های rs1050450، rs4673 و rs13306294 و فعالیت گلوتاتیون پراکسیداز-1 در بیماران مبتلا به تنگی عروق کرونر در شهر تهران

http://yafte.lums.ac.ir/article-1-2177-fa.html

مقدمه: تنگی عروق کرونری یکی از عوارض ابتلا به آترواسکلروزیس بوده که می‌تواند منجر به کاهش اکسیژن‌رسانی به بافت قلب و سکته قلبی شود. پلی‌مورفیسم rs1050450 یکی از پلی‌مورفیسم‌های مهم آنزیم گلوتاتیون پراکسیداز-1 (GPx1) بوده که می‌تواند فعالیت آنتی‌اکسیدانتی آن را تغییر دهد. همچنین دو پلی‌مورفیسم آنزیم NADPH اکسیداز، پلی‌مورفیسم‌های rs4673 و rs13306294، پیشنهاد شده است که نقش مهمی در عملکرد این آنزیم دارند. در این مطالعه ما به بررسی پلی‌مورفیسم‌های rs1050450، rs4673 و rs13306294 در بیماران با تنگی عروق کرونری پرداختیم. مواد و روش­ ها: 190 نفر از بیماران آنژیوگرافی شده به دو دسته بیمار (114=n) و کنترل (76=n) تقسیم شدند. پس از نمونه‌گیری پروفایل لیپیدی و مکان‌های پلی‌مورفیک به ترتیب با روش‌های روتین آزمایشگاهی و تست RFLP-(ARMS)  PCR ارزیابی شدند. یافته­ ها: در این مطالعه تفاوت معناداری بین سطوح پلاسمایی LDL و درجه تنگی عروق دیده شد. ما همچنین بین پلی‌مورفیسم rs13306294 و تنگی عروق کرونری رابطه معناداری مشاهده کردیم اما زمانی که سن، جنس و BMI لحاظ شد تفاوت معناداری بین پلی‌مورفیسم‌های rs1050450، rs4673 و rs13306294 و تنگی عروق کرونری مشاهده نشد. بحث و نتیجه­ گیری: پلی‌مورفیسم‌های rs4673، rs13306294 و rs13306294 نقشی در پیشرفت تنگی عروق کرونری ندارند.

Title: اثر نوع تمرین بر نشانگران آپوپتوز هپاتوسیتی در رت‌های تغذیه شده با غذای پرچربب

http://yafte.lums.ac.ir/article-1-2540-fa.html

مقدمه: اثرات ضد التهابی و ضد آپوپتوتیک فعالیت بدنی مشخص شده است و اثربخشی آن وابسته به شدت، مدت و نوع می‌باشد. چون چاقی به عنوان التهاب خفیف مزمن در نظر گرفته می‌شود، منطقی است آسیب‌های دژنراتیو بافتی به ویژه در کبد به عنوان مرکز متابولیسم رخ دهد. هدف مطالعه حاضر، بررسی اثر دو نوع تمرین هوازی (استقامتی و تناوبی شدید) بر نشانگران آپوپتوز هپاتوسیتی در رت‌های تغذیه شده با غذای پرچرب بود. مواد و روش­ها: در یک کارآزمایی تجربی، 28 سر رت نر نژاد ویستار به صورت تصادفی در چهار گروه کنترل نرمال، کنترل- غذای پرچرب، غذای پرچرب-تمرین استقامتی، غذای پرچرب-تمرین شدید تناوبی قرار گرفتند. در مرحله اول به مدت 13 هفته آزمودنی‌ها غذای پرچرب دریافت نمودند. پس از آن به مدت 12 هفته، هفته‌ای 5 جلسه به تمرین پرداختند. در پایان دوره، پس از بیهوشی بافت کبد جهت سنجش بیان ژن‌های مسیر آپوپتوز برداشته شد. یافته ­ها: تمرین با شدت بالا موجب افزایش معنی‌دار بیان ژن BAX شد (01/0P=)، در حالی که اثر معنی‌داری بر بیان ژن Bcl-2 نداشت (06/0P=). تمرین شدید تناوبی در مقایسه با تمرین استقامتی با شدت متوسط موجب افزایش در نسبت 2 BAX/Bcl- شد. الگوی تغییرات Caspase-9 نیز مشابه تغییرات نسبت BAX/Bcl-2 بود. بحث و نتیجه ­گیری: نتایج نشان داد، اثر فعالیت بدنی بر مسیر آپوپتوز هپاتوسیتی وابسته به نوع و شدت آن می‌باشد. لذا توصیه می‌گردد از تمرینات استقامتی با شدت متوسط برای حفاظت کبد از آسیب‌های ناشی از تغذیه با غذای پرچرب استفاده شود.

Title: بررسی رابطه بین افسردگی و عزت نفس با عملکرد تحصیلی در دانشجویان دانشگاه آزاد اسلامی واحد خرم‌آباد

http://yafte.lums.ac.ir/article-1-2446-fa.html

مقدمه: عملکرد تحصیلی در هر جامعه نشان دهنده موفقیت نظام آموزشی در زمینه هدف‌یابی و توجه به رفع نیازهای فردی است. بنابراین نظام آموزشی را زمانی می‌توان کارآمد دانست که عملکرد تحصیلی فراگیران آن در دوره‌های مختلف دارای بیشترین و بالاترین رقم باشد. مواد و روش ­ها: روش تحقیق توصیفی از نوع همبستگی بود. جامعه آماری این پژوهش شامل دانشجویان فنی-مهندسی و علوم انسانی دانشگاه آزاد اسلامی واحد خرم‌آباد مشغول به تحصیل در سال تحصیلی 95-1394 به تعداد 7761 نفر بود. حجم نمونه با استفاده از جدول کرجسی و مورگان، 351 نفر برآورد شد و به روش نمونه‌گیری تصادفی طبقه‌ای نسبی انتخاب شدند. ابزار گردآوری داده‌ها شامل پرسشنامه عزت نفس کوپر اسمیت (1967)، پرسشنامه افسردگی بک (1996) و معدل دانشجویان در سال تحصیلی 95-1394 بود. جهت تحلیل داده­ها از شاخص‌های فراوانی، درصد فراوانی، میانگین و انحراف معیار و آزمون‌های ضریب همبستگی پیرسون و رگرسیون چندگانه به روش گام به گام استفاده شد. یافته ­ها: نتایج نشان داد بین افسردگی با عملکرد تحصیلی دانشجویان مورد مطالعه، رابطه منفی و معناداری وجود دارد و بین عزت نفس و ابعاد آن (عزت نفس عمومی، عزت نفس خانوادگی، عزت نفس اجتماعی و عزت نفس تحصیلی) و عملکرد تحصیلی رابطه مثبت و معناداری وجود دارد. هم‌چنین بر اساس نتایج تحلیل رگرسیون، عزت نفس تحصیلی، عزت نفس خانوادگی و افسردگی 08/0 از واریانس نمرات عملکرد تحصیلی دانشجویان شرکت کننده در پژوهش را تبیین می‌کنند. بحث و نتیجه­ گیری: با توجه به نتایج مطالعه حاضر مشاهده شد که میان افسردگی و عملکرد تحصیلی دانشجویان، رابطه منفی و معنادار و نیز میان عزت نفس و عملکرد تحصیلی رابطه مثبت و معناداری وجود دارد.
